# *‘Nurture the sprouting bud; do not uproot it’*. Using saving groups to save for maternal and newborn health: lessons from rural Eastern Uganda

**DOI:** 10.1080/16549716.2017.1347311

**Published:** 2017-08-18

**Authors:** Elizabeth Ekirapa-Kiracho, Ligia Paina, Rornald Muhumuza Kananura, Aloysius Mutebi, Pacuto Jane, Juliet Tumuhairwe, Moses Tetui, Suzanne N Kiwanuka

**Affiliations:** ^a^ Makerere University School of Public Health, Department of Health Policy Planning and Management, Kampala, Uganda; ^b^ Department of International Health, Johns Hopkins Bloomberg School of Public Health, Baltimore, MD, USA; ^c^ Reproductive Health Division, Ministry of Health, Uganda; ^d^ Department of Public Health and Clinical Medicine Sweden, Umeå University, Epidemiology and Global Health Unit, Umeå, Sweden

**Keywords:** MANIFEST - Maternal and Neonatal Implementation for Equitable Systems Study, Saving groups, maternal, newborn, birth preparedness, implementation science, health insurance

## Abstract

**Background**: Saving groups are increasingly being used to save in many developing countries. However, there is limited literature about how they can be exploited to improve maternal and newborn health.

**Objectives**: This paper describes saving practices, factors that encourage and constrain saving with saving groups, and lessons learnt while supporting communities to save through saving groups.

**Methods**: This qualitative study was done in three districts in Eastern Uganda. Saving groups were identified and provided with support to enhance members’ access to maternal and newborn health. Fifteen focus group discussions (FGDs) and 18 key informant interviews (KIIs) were conducted to elicit members’ views about saving practices. Document review was undertaken to identify key lessons for supporting saving groups. Qualitative data are presented thematically.

**Results**: Awareness of the importance of saving, safe custody of money saved, flexible saving arrangements and easy access to loans for personal needs including transport during obstetric emergencies increased willingness to save with saving groups. Saving groups therefore provided a safety net for the poor during emergencies. Poor management of saving groups and detrimental economic practices like gambling constrained saving. Efficient running of saving groups requires that they have a clear management structure, which is legally registered with relevant authorities and that it is governed by a constitution.

**Conclusions**: Saving groups were considered a useful form of saving that enabled easy acess to cash for birth preparedness and transportation during emergencies. They are like ‘a sprouting bud that needs to be nurtured rather than uprooted’, as they appear to have the potential to act as a safety net for poor communities that have no health insurance. Local governments should therefore strengthen the management capacity of saving groups so as to ensure their efficient running through partnerships with non-governmental organizations that can provide support to such groups.

## Background

Skilled attendance at delivery can lead to significant reductions in maternal mortality [1]. According to the demographic health survey for Uganda, 438 women of 100,000 die as a result of pregnancy-related complications [2]. Failure to access skilled delivery care services has been linked to three main delays – delay in seeking care, delay in reaching care and delay in receiving appropriate care [3]. Access to cash is one of the factors that contribute to these delays [4–6]. Therefore, households are often encouraged to prepare for birth by saving money that can be used during pregnancy and at the time of birth [7,8]. Access to cash through savings is also beneficial for meeting other personal needs, personal emergencies such as illness and accidents as well as life cycle needs, such as marriage and death [9,10]. In spite of the above-mentioned benefits of saving, many people do not save. World-wide, 22% of people were reported not to have saved in a financial institution in the past 12 months, while 77% of adults who earn less than $2 a day did not have an account in a formal financial institution [11]. Similarly, Uganda has very low saving rates. Only 5.2% of Ugandans were reported to be saving [12]. Furthermore, work done by the Uganda Economic Policy Research Center in 2013 estimated that about 15% of Ugandans are excluded from any form of financial services [12].

Ugandans save using a combination of formal and informal methods. The formal methods include financial institutions such as banks, semi-formal financial institutions such as microfinance institutions and Savings and Credit Co-operatives (SACCOs). These formal saving methods are often utilized mainly by the middle and high class [9,12–14]. Those in the lower classes tend to prefer informal methods that include Rotating Savings and Credit Associations (ROSCAs), Accumulating Savings and Credit Associations (ASCAs), home banks, money guards, deposit collectors, deposits with suppliers and savings in kind [9,12–14]. In some cases these groups complement local financial services such as banks and microfinance institutions, while in other cases they target only those that are excluded from these services [15–17]. These informal methods are preferred by the poor, because they are often located close to them, are flexible, based on savings rather than credit, and have a range of products that meet the needs of the poor, such as small loans, acceptance of daily small deposits of money and uncomplicated quick procedures for borrowing money [9]. However, many of these informal services are provided through mutual trust, hence it is often difficult to seek legal redress when the social contracts are broken and money is lost or misused. They therefore face several challenges that include loss of money from theft, defaulters, misuse, loss of assets, poor governance and management. Furthermore, this informal saving limits their ability to benefit from high interest rates and loans [9,18].

One of the informal saving methods that are being used increasingly in developing countries are ASCAs, referred to in this paper as saving groups [15,19,20]. Saving groups are also seen as a means of enhancing social cohesion and hence social capital, a means of promoting women’s leadership, improving gender relations, as well as community and economic development [17,21]. These saving groups are typically comprised of about 22 to 30 self-selected individuals who save an agreed amount of money on a regular basis. Members of saving groups can borrow out of the collection, agreeing to repay the money later at a small interest. The income earned from the interest is kept within the group and shared out among members at the end of a pre-defined cycle according to the proportion of their initial contributions[15–17,19,22]. Some saving groups also have a social fund, which is a form of limited self-managed insurance to which all the members contribute a set amount and can get interest-free loans for emergencies [15–17,22]. A detailed description of the saving groups in the MANIFEST study has been provided in one of the papers in this supplement [22].

As alluded to earlier, savings are useful for meeting personal emergencies such as illness and life cycle events such as birth [9]. Money from saving groups has also been used to meet the cost of illnesses, such as malaria, to meet the cost of transportation to health facilities, or to improve access to information on topics such as malaria prevention and HIV [17,22]. In Pakistan, women who saved with community-based saving groups (CBSGs) were more likely to utilize maternal health services, such as antenatal care and delivery care, than those who did not belong to CBSGs [23]. However, the results of a review of seven randomized controlled trials surprisingly did not show increased expenditure on children’s health [20]. One of the explanations proposed included a substitution effect in which the household still spends on health, but gets this money from the saving groups, in a more timely and less shameful manner (e.g. begging) [20]. Due to the inconclusiveness of the results of the trial, the authors recommended further research on saving groups so as to build a better understanding of how and why they work, and for whom [20].

It is against this backdrop that a team of researchers from Makerere University School of Public Health, through the MANIFEST study [8], explored the potential of a participatory multisectoral project to increase the utilization of maternal health services and newborn care practices. The project employed a quasi-experimental design with an intervention arm and a comparison arm in three rural districts in eastern Uganda where the predominant occupations include crop farming, animal rearing and petty trading. The project had two main components: community empowerment for comprehensive birth preparedness, and health provider capacity-building. The community mobilization and empowerment component comprised (1) provision of health education about maternal and newborn health through home visits by community health workers, community dialogues, radio spots and radio talk shows; (2) initiatives to improve access to finances through the promotion of household savings; and (3) initiatives to improve access to transport services through the use of locally available transporters (boda bodas – motorcycles). The health provider capacity building component consisted of (1) refresher training in maternal and newborn health, (2) mentorship and support supervision, (3) purchase of basic equipment, and (4) Recognition of best-performing health workers. Savings for maternal and newborn health was enhanced by promoting various models of saving, which included the use of saving groups, in addition to individual saving through home banks and saving in kind, for example through the purchase and sale of easily convertible assets, such as livestock. Additional details about the intervention are available in the study design paper [8]. Key characteristics of the saving groups are further described in Mutebi et al. [22], while information about how we worked with the saving groups is provided in the methods section of this paper.

Due to the paucity of literature around the use of saving groups for maternal health, in this paper we describe saving practices for maternal health, factors that facilitate and constrain saving for maternal health through saving groups and lessons learnt in the process of supporting communities in the MANIFEST implementation areas to save for maternal health using saving groups. The influence of saving on utilization of maternal health services and the influence of other components of the intervention have been described in the other papers that are included in this supplement [24,25].

## Methods

### Study design

This was a qualitative study comprising focus group discussions (FGDs), key informant interviews (KIIs) and review of project documents and meeting notes. It was conducted across three districts (kamuli, Kibuku and Pallisa).

The section below describes how we worked with saving groups to enhance saving for maternal health.

### The process of harnessing saving groups for maternal and newborn health

**Figure 1.**
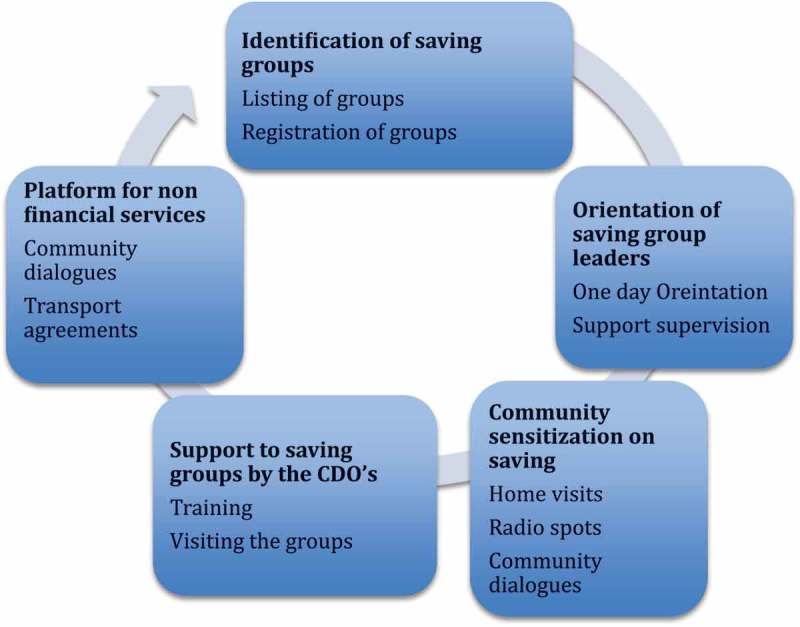
Process of setting up maternal health component in existing and new saving groups.

The process of engaging with savings groups was achieved through five main activities that have been summarized in Figure 1. The activities were repeated every quarter except for the orientation which was done only once at the beginning of the study.

The activities included the following:

1. *Identification of saving groups*: the first step in the process was to identify existing saving groups. Use of existing saving groups as natural groups, which can sustain themselves, was considered important for the sustainability of saving groups. Existing saving groups were listed by the community development officers and the village health teams (VHTs). The process of listing the groups helped the team to characterize them and determine their capacity to support health. In addition, it ensured that the VHTs had a list of existing saving groups to which they could direct women when they went for home visits.
2. *Orientation of the saving group leaders*: a one-day orientation of saving group leaders was done by the research team and community development officers (government officials responsible for providing support to saving groups). One saving group leader was invited from each of the villages in the intervention area. The orientation equipped them to perform their roles, which included: encouraging the community to join saving groups and to save for maternal health, adding a maternal health component to their saving group, ensuring that the saving group adhered to the recommended management practices, identifying a transporter and making an agreement with him to transport pregnant women to the facility, as well as lending pregnant members from their groups money beyond what they had saved if they had an emergency.
3. *Community sensitization*: the sensitization was done through radio messages, home visits by community health workers and community dialogues. All these activities emphasized the importance of saving for maternal and newborn health and encouraged community members to save using saving groups and other methods.
4. *Provision of support to saving groups*: management of the saving groups was strengthened through support provided by the community development officers who received a two-day training to strengthen their capacity to support the saving groups. The support that they provided to the groups comprised supervision visits and quarterly training. The training focused on making constitutions, managing group dynamics, financial management and record keeping as well as income generation.
5. *Saving groups as a platform to provide non-financial services*: the saving groups were used as a platform to provide other non-financial services, such as transport services and health education about maternal and newborn health services. The groups were encouraged to make formal and informal agreements with transporters so that these transporters could transport women to the health facilities for routine services, such as antenatal care and delivery, as well as for emergency services, such as referrals for patients with obstructed labour. Table 1 provides a summary of the groups that formed maternal and child health accounts as well as those who formed agreements with transporters.

**Table 1.** Saving groups with maternal and child health accounts and transport agreements.

Item	Kamuli	Kibuku	Pallisa	Total
Number of saving groups (new and old)	279	415	219	913
Saving groups with signed transport agreements	47	128	88	263
Percentage of saving groups with signed transport agreements	17	31	40	29
Number of saving groups with maternal and child health accounts	61	95	148	304
Percentage of saving groups with maternal and child health accounts	22	23	68	33

### Data collection methods and tools

Eighteen key informant interviews (KIIs) and 15 Focus Group Discussions (FGDs) were held in the intervention and comparison areas in the three districts. The 18 key informants (12 in the intervention arm and six in the comparison arm) were selected purposively, either because they were involved in the implementation of the study and therefore knowledgeable about the study (three CDOs, one super village health team member (VHT), four chairpersons and treasurers of saving groups, two subcounty chiefs, three health assistants) or because they were local opinion leaders (three local council chairpersons and two secretaries for health) and were, therefore, in position to share their observations and perceptions about the savings component of the project and its influence on the community.

Nine FGDs were done in the intervention and six in the control areas. Six focus groups were held with men of reproductive age and nine were held with women of reproductive age. Each focus group had six to 12 participants. The focus group participants were selected with the help of the local council chairperson, who is a gatekeeper in the community. The members of the female focus groups were women of reproductive age who had given birth within one year prior to the data collection period, while participants for the male focus groups were men whose wives had given birth within one year prior to the data collection.

A key informant guide and FGD guide was used to collect data from the key informants and the focus group participants. The information collected focused on issues such as saving practices for maternal health, the experiences of the respondents with saving groups, benefits of joining saving groups, male involvement in saving groups, challenges encountered in saving groups and perceptions about the savings component of the project (with a focus on areas where it worked well and where challenges were noted, as well as suggestions for improvement). The FGDs and KIIs were conducted by a total of four facilitators who had experience in collecting qualitative data. Data extraction guides were used to extract data from the project reports and meeting notes.

### Data management and analysis

The interviews were recorded using a digital recorder and then later transcribed verbatim and reconciled with the notes taken during the interviews. The analysis was done manually using thematic analysis. The transcripts were first read and re-read to allow familiarization with the data. A coding frame was then developed in line with the research questions. The transcripts were then coded and both deductive and inductive coding approaches were utilized. The deductive approach to coding was based on the key issues that we sought to answer in relation to our study objectives, while the inductive approach allowed us to include new issues that emerged as we read the data. After that, categories were derived and themes generated from the data. The categories that were identified when looking at saving practices included individual saving methods and group saving methods. The categories that emerged when looking at factors that facilitate or constrain saving included individual factors, benefits of joining saving groups and management-related factors. When looking at lessons learnt, we used the five stages as categories and then identified themes within each of them. Interpretations and arguments were then drawn from the data in relation to the objectives and presented as text. The project documents were reviewed and key lessons were synthesized out of them and used to draw key lessons which were presented as text and a matrix.

### Ethical considerations

Written informed consent was obtained from all key informants, while verbal informed consent was sought from focus group participants. Ethical approval was provided by the Makerere University School of Public Health Higher Degrees Research and Ethics Committee (HDREC-152) and the National Council of Science and technology (UNCST-HS1399).

## Results

In this section, we present results about saving practices for maternal health, factors that facilitate or hinder saving with saving groups and lessons learnt from the process of harnessing saving groups for maternal health.

### Saving practices for maternal health

Saving practices for maternal health are not different from the other methods used to save for other health or non-health issues. They comprised two main methods; group savings through the saving groups and individual saving.

The respondents who save using saving groups reported that they saved for maternal and child health, especially for transport to and from the health facility during pregnancy and at the time of delivery. The money saved for maternal health under the saving groups is readily available because it is not lent out, hence it can be withdrawn at any time in case there is a problem. This was captured in quotations such as those below.

'Recently I lost my uncle and my husband spent all the money that he had and I’m now expecting any time, so I went and withdrew some money from my savings (from savings group) and did some shopping for the newborn baby.' (Focus group discussion with women in the intervention area)

'At least men are also aware that saving is not only for women; it’s for both, because these days men can give their women money that, ‘you take this money to the saving group’.' (Key informant interview with CDO in the intervention area)

Individual saving comprised saving money at home (home banking) and the storage of items such as goats, chickens or even food crops that were sold off to obtain cash when it was needed (saving in kind). This form of saving was common for those who are peasants; it was especially common in the comparison area.

'In this community, most people – like now tomorrow Monday is market day – they calculate and say going to and from Butebo is 6000/- so what a man decides is to tell a woman that ‘go and uproot cassava, dry it’, if she is to go to the facility, she will find a trader and sell the cassava and that is the money she will use. That is the only way money is saved by the biggest population here who are peasants.' (Focus group discussion with men in the comparison area)

Some respondents said that they save their money individually until they require it. This was reported in the FGDs from both the intervention and comparison area as noted below.

'I have got this money; keep/save it, even if it’s 1000/ = he gives me and I save, until I get like 30,000/ = . I go there once and buy what I want and now, like this other money is waiting for me to deliver … if you agree, he can give you and you keep/save it and when it reaches time for delivery you have the money.' (Focus group discussion with women in the intervention area)

'Ok me I teach and I am paid for a month. I use this money to prepare myself when I am pregnant. I don’t vomit or need a special diet, so this helps me to save money.' (Focus group discussion with women in the intervention area)

From the focus group discussion with men, it was indicated that some men are also involved in preparing for birth by putting aside some money that can be used at the time of delivery. One of the group members narrated:

'Responsible men have been putting some money aside to prepare for birth, therefore that money is only accessed at the time of delivery. Whatever the condition will be which requires money, you can’t touch that money, because it was not for that purpose.' (Focus group discussion with men in the comparison area)

### Factors that encourage saving with saving groups

During the FGDs, participants identified several factors that influenced the joining of saving groups as a method of saving for maternal health and other issues. We categorized these under three main groups: individual factors, benefits of belonging to saving groups and management of saving groups.

The individual factors included awareness about the importance of saving and low incomes.

### Awareness about the importance of saving

The savings culture was not novel within the community, because they have been doing it with the goal of sharing the money during festive holidays. Savings allowed people have access to money to meet some of their needs during festive seasons. However, communities had not been sensitized about saving for health. The majority of participants in focus group discussions indicated that increased awareness about the importance of saving for maternal and newborn health contributed to families saving for this purpose. These issues are illustrated in the quotes below.

'Again we are now saving for women; this program has brought us from far, because we did not know anything about saving for health … Then I was told that when your wife is pregnant, that is the money they use for transport to go to the health facility and when delivery time comes, maybe at night, there is a transporter who will take her to the health facility. So we save for them (their women).' (Focus group discussion with men in the intervention area)

One of the methods recommended to increase awareness about the importance of savings, was sensitization through local leaders.

'What I see is for government to pass through politicians, like local councils, to sensitize communities on savings and also mobilizing meetings where they can get information from, but targeting specifically those men who don’t want to participate.' (Focus group discussion with men in the intervention area)

### Low incomes

Low incomes was mentioned in the KIIs and FGDs, for both men and women, as one of the main factors that influence savings for maternal and newborn health. Families which are affluent normally do not save because they can afford to pay the bills, whereas most families which have low incomes are reported to have saved so that they can ensure that the money is available when it is required.

The benefits of belonging to saving groups encouraged people to join the groups.

### Easy access to loans

One of the benefits that encouraged the community members to join saving groups was easy access to loans. The conditions for borrowing were usually not as stringent as they are in the banks. Money that was borrowed from the saving group could be used for several purposes that included meeting transport costs, paying school fees, starting a business or even paying hospital bills. This is reflected in the quotes below.

'We had one incident one day in Gogonyo HC III, whereby the woman failed to deliver and the VHT rang the group members to contribute money for fueling the vehicle, which was at the facility at that time. The VHTs brought the form from the nurse for members to sign and the money was released, since the mother also used to save. So they fueled the vehicle and the mother was transported to Pallisa hospital.' (Key informant interview with saving group leader in intervention area)

'Another thing which has encouraged people to join saving groups is that if someone has any problems, he will run to the saving group to borrow money … to sort out his problem. But before these saving groups existed, it was very difficult to get money anywhere in case of any problem – may be the child has been sent away from school for school fees – a person will end up selling any asset he/she has in his house.' (Key informant interview with Saving Group Leader Intervention)

### Safer custody of money

Community members also joined saving groups because the saving groups enabled them to keep their money until they needed it. This money enabled the households to buy additional birth requirements, such as gloves, baby clothes, etc.

'For me I have benefited because I have managed to save and prepare in case my wife reaches delivery and even to take care of her after delivery. I have been able to secure facility requirements, like gloves, mackintosh and the like, in time.' (Focus group discussion with men in the intervention area)

Furthermore, the saving groups also allowed them to save for non-health-related activities, such as the payment of school fees, new clothes and festive seasons expenses.

'Another thing which is making people join saving groups is the public days, like Uhuru day, Christmas day. During this season, the groups will open up their saving boxes, then they divide their money according to what they saved and after they even divide the interest they got from loaning out their money. And once this money is divided, it changes people’s ways of living. Some people buy goats, new clothes, food for those big days and so many other things. But after the money is divided, they always leave some for starting again another year.' (Saving group leader in the intervention area)

### Flexible savings

One of the facilitating factors related to management of the groups was flexible saving. This allowed members of different socioeconomic status to join saving groups. Many saving groups had a minimum amount and a maximum amount that members were expected to save. The minimum amount was often very low. Others allowed members to save any amount that they thought they could afford to pay, as long as they saved regularly.

'(adding money) is a very hard thing because it depends on the season and it is one thing that can chase away members in the group when you are telling them that bring this amount of money that they can’t get. That’s why we left it open so that everybody can contribute what they can.' (Saving group leader in the intervention area)

### Factors that constrain saving using saving groups

Despite the cited advantages, there are some community members who do not save for maternal and newborn health. Whereas some of the factors were individual, others were related to the management of saving groups.

### Poverty

Poverty was the commonest factor that was reported to constrain both individual and group saving practices. It was noted that often people did not save because they had nothing to save (no access to cash).

'I think some men fail, not because they don’t want to help their women, but you see we are not all the same. Some don’t have (money) so you cannot keep insisting that ‘I want this and that’ when you also know that he does not have the money. The only solution is to get used to the situation and life continues.' (Focus group discussion with women in the intervention area)

### Lack of prioritization of health issues

It was also noted that some males do not participate in saving because they prioritized other things over saving for health. They would prefer to attend political rallies or to join their friends at drinking gatherings rather than participate in a saving group meeting. Other women also expressed concern that joining saving groups can potentially make spouses neglect their responsibility to provide and that they would squander money in unproductive gambling. Polygamous men also tended to abandon their responsibilities and to leave issues such as saving for maternal health to their wives.

'These days there is ‘ludo’ (a gambling game) and I think most men take their money to play ludo instead of saving. At times when he realizes that you [woman] are saving, he will not bother himself again ...' (Focus group fiscussion with women in the intervention area)

'I have seen from other communities out there (that) when a man gets more than one wife he ignores his responsibility and leaves the entire burden on the woman, so every woman has to work hard and look after herself as the man takes care of himself.' (Focus group discussion with men in the intervention area)

### Selfishness

A minority do not save because they felt that when they save, they give other people their money and improve other people’s welfare, so they decide not to join a saving group.

'People will say, ‘how can I give my money to a group for other people to use for educating their children?’' (Key informant interview with saving group leader in the intervention area)

### Poor management of saving groups

Previous experiences of saving group leaders disappearing with the group’s money caused community members to lose trust in some of the savings groups.

'You may have picked yourselves … we elect chairmen, treasurers – you may be there and he has taken off with the money. So this also stops someone from joining the group when you hear that (someone) left the other side (another saving group) someone loses heart.' (Focus group discussion with women in the intervention area)

### Dissatisfaction with credit modalities

Some community members did not join saving groups because of the high interest rates when members want to borrow money, while others feared that their property would be detained if they failed to pay back the money.

'At times she fears and you also know that other groups (savings) can detain those with debts. … or they come and confiscate things like that and we also put a security like a cow or fridge when you are going for a loan and (if) you fail to pay that is the security they take to pay back their money. So others fear because of that reason.' (Key informant interview with saving group leader in the comparison area)

### Lessons on harnessing saving for maternal and newborn health

There are several saving groups already in existence in these districts. However, most groups do not traditionally save for health. Although informal, many of these have built structures of management and leadership and trust within the community. Registration of saving groups and availability of group constitutions is vital for securing legal redress whenever fraud occurs within the group.

Illiteracy, lack of management expertise, poverty and lack of investment options hinder the growth of saving groups. Table 2 presents a summary of barriers, facilitators and lessons learned.

**Table 2.** Lessons learned from the process of promoting saving groups for maternal and newborn health.

	Benefits	Challenges	Lessons
Identification of existing saving groups	Pre-existing saving groups stable in terms of membership, management and saving practices. There are multiple partners promoting saving at community level. Whereas most groups are dominated by women, the men also had their own groups and some had both men and women.	Some areas do not have pre-existing groups and groups need to be established. Pre-selection of members results in inequity. Previous bad experiences hinder joining existing groups. Male membership in female groups introduces gender power dynamics, making the women less secure of their rights and the security of their money.	Pre-existing groups are more sustainable and are a resource for mentoring new groups. Existing partners can reduce inequity by supporting those who are unable to join and save. Allow the community members to set their own criteria for joining saving groups.
Orientation of the saving group leaders Registration Constitution Management of funds	Training of group leaders fosters confidence in members. Registration of saving groups is important for seeking legal support in case defaulting borrowers Constitutions with guidelines for membership, leadership, collection and utilization of funds are important for guiding their operations. Groups are able to generate substantial amounts of money within a reasonable period of time	Illiteracy restricts building financial management skills Small groups tended not to register. Illiteracy is a barrier for developing and implementing constitutions. Lack of focus on health. Large funds attract fraud and theft.	Training should not be one-off but rather continuous. Small groups need to be supported to register by making procedures simple and costs affordable. Language used in constitution needs to be contextualized and simplified. Orient leaders on how to incorporate health within constitutions. Groups need to be supported to secure bank accounts or mobile money accounts.
Provision of support to saving groups	Investment of group savings helps generate interest and more income to the groups. Properly managed groups are likely to grow and to be sustained.	Many groups don’t invest their money. Poor selection of investments can lead to financial losses and loss of trust.	Provide support through existing structures like community development organizations or NGOs.
Saving groups as a communication platform	Groups, many of which are predominantly female, provide opportunities for educating members on various health issues. Saving groups enhance social cohesion beyond health.	Members are not health experts, making the group vulnerable to misinformation. They often exclude the most vulnerable.	There is a need to provide information and education materials to groups. Invite health expert to educate in some sessions or incorporate a VHT in each group.

## Discussion

In this discussion we share how community saving practices influence their choices of savings and how we can support formal saving through saving groups, propose support mechanisms for those who do not save and how we can harness the use of saving groups for maternal health. Like the saying goes, ‘there is more than one way of skinning a cat’. Being part of a saving group is not the only mechanism for saving towards supporting maternal health. Ultimately, the goal of saving is to ensure that resources are available to contribute towards health needs.

### Factors that encourage saving for maternal and newborn health

Awareness about the importance of saving as part of the preparation for birth was one of the factors that spurred the community towards saving. Knowledge about the importance of birth preparedness is important for improving birth preparedness and subsequently improving maternal service accessibility especially when presented as part of a package of interventions that reduce the three delays to seeking maternal and newborn health services [3,26–28]. The transmission of knowledge about birth preparedness by targeting family members and male partners was therefore able to encourage more men to start saving for maternal health. As decision-makers and family heads it is indeed critical that men get involved in birth preparedness activities [26,27]. This is likely to shorten delays in making decisions to seek care and to reach care because lack of cash is one of the factors responsible for this delay [3,7,28].

The other factors that encouraged people to save were linked to the benefits of saving, which allowed them to keep their money until they required it. Consequently, they were able to use the money to meet needs such as purchasing baby clothes, transportation to the facility and even non-health needs such as school fees. Another major benefit was that the saving groups allowed them easier access to loans than other financial institutions, which tended to have collateral that was beyond their reach. This money was sometimes used for emergency referrals; saving with saving groups therefore has the potential to effectively reduce one of the critical delays that often results in failure to receive timely obstetric emergency care [3,28].

### Factors that constrain saving for maternal and newborn health

In spite of the benefits of saving, our work showed that some people still do not save for health. For example, some men still tended to prioritize alcohol over maternal health-related issues. Implementers of maternal and newborn health programmes should therefore continue to sensitize households, so that they can prioritize and set aside some money for birthpreparedness.

Poverty was another factor that barred people from joining saving groups. When saving groups decide on a fixed amount of money, those who cannot afford that amount of money are not able to join the group. This leads to exclusion of the most poor from saving groups. This is in keeping with other studies that have reported lack of acess to cash and high fees as reasons that were given for not saving in SACCOs [18].

To counter this problem, some of the groups used a flexible system that allowed individuals to save what they were able to afford whenever payment was required. Hence, while some members were able to pay, for example, sh5,000 per week, others were free to pay only sh1,000 per week. Other programmes like ‘Saving for Change’, supported by Oxfam, also encouraged people to save what they can afford, rather than fixed amounts [16]. Alternatively, saving groups could also be given subsidies, which would allow them to recruit disadvantaged populations such as pregnant teenage mothers or the very poor as members. Such adolescents can be encouraged to form their own saving groups where they feel accepted. They would, however, need to be supported with initial income to invest. The government of Uganda recently started a youth livelihood programme that provides an opportunity for young people to receive financial resources that they can invest [29]. Matching of funds saved has also been suggested as a method of encouraging and rewarding saving [14]. These concepts can be encouraged as a way of society helping those in dire need. Furthermore, many saving groups have social funds, which are often used to meet needs such as emergencies and burial fees and can be used to support the vulnerable who are unable to save even such small amounts [17,19].

### Lessons learnt

Our work also provides important lessons for increasing enrollment of the poor into insurance schemes. Many developing countries have tried to use community-based health insurance to increase access to services [30–33]. Some of the factors that have led to low enrolment and hindered the growth of these community-based health insurance schemes include inability to pay premium and rigid payment mechanisms [30–33]. Increasingly, countries are now turning towards health insurance schemes that cater for the poor by asking central or local governments to pay their premiums [34–36]. In developing countries, the latter option may not be very viable because of the limited budgets in these governments; therefore, innovative ways of encouraging increased enrolment of the informal sector into community-based or social health insurance schemes is critical if social protection is to be provided for the poor [31,34–36]. Saving groups provide an opportunity for community members to accumulate money, which can then be used to pay premiums for health insurance [37]. Secondly, as noted above, some saving groups have flexible payment arrangements, which could be emulated by community-based health insurance schemes.

Widespread application of these suggestions would, however, require governments that have abolished health user fees to be willing to allow the community to contribute funds for health service delivery in the public sector, which is the main provider of services in many developing countries. Work done by Dakoye et al. in Uganda [38] showed that there was willingness to pay for services. Eighty-three percent of the people interviewed were willing to pay some money in public facilities if the quality of services improved. The willingness was higher among the poorer quintiles that often suffer from catastrophic expenditure [38]. The amount they were willing to pay, however, was rather low, which emphasizes the fact that this form of payment can only complement what government provides rather than meet the whole cost of care.

MANIFEST utilized both new and existing saving groups. Pre-existing groups were favored above newly established ones, because members had built trust and had an organized way of managing their funds [16]. Having a constitution, bank account and knowledgeable, trusted, transparent leadership reinforced members’ confidence to save with the group. This is particularly important because lack of trust in the group was one of the factors that hinder saving with SACCOs [18]. Furthermore, the use of existing saving groups allows the utilization of natural groups that can be sustained, because they already have a reason that brings them together and they often have stable saving patterns and membership. Flexibility in such groups to allow them to add other components of saving is therefore important. For example, in the implementation area where we were working, existing groups added a maternal health component to allow them to save for maternal health. However, these groups need to be supported and regulated to have clear management structures, which includes a management team that is responsible for the management of the group, a constitution that guides their operations, safe mechanisms for ensuring the security of savings such as banking, as well as registration with local governing authorities. Without such structures, the money saved could be at risk [14,18,22].

Many of the groups in our implementation area were led by community members who had limited experience in financial management and yet the existing support network through the community development officers was initially non-functional because of lack of facilitation [22]. The groups that were supported by NGOs (Village Loans and Saving Associations) in our setting were better managed. Partnerships between government and such organizations can enhance the support given to saving groups. However, programmes elsewhere have found models where the community members are trained to be village agents cheaper than where the community relies on assistance provided by NGOs [23].

Another area that warrants urgent attention is the need for the groups to invest their income so as to allow for growth. At the start of the project, many of the groups did not have significant sums of money because they used to lend out most of the money that they collected. As their savings grew they found that they could not lend out all the money and yet they did not know how to invest the money. Saving groups therefore need guidance that can enable them invest their income in profitable viable ventures [22]. Such actions are critical for the generation of additional household income, and can contribute to the ability of households to have sufficient income to meet their immediate needs. Other authors have also noted the importance of training saving groups to invest their income [20].

### Strengths and limitations

One of the strengths of this paper is that it used qualitative methods to capture the participants’ own perceptions of saving groups while highlighting the factors that encourage them to join saving groups and those that hinder them from joining saving groups. This can be useful for guiding initiatives aimed at enhancing saving for maternal health through saving groups. Although we acknowledge that both formal and informal methods of saving are important, one limitation of this paper is that it focused mainly on exploring the use of saving groups as a way of securing resources for maternal health. Another limitation is that we did not have longitudinal data about money saved through the saving groups and through individual savings. This would have allowed us to analyse the use of saving groups more extensively. We do believe that our findings are generalizable to rural settings that have a similar context to the districts studied. Lastly, our study was done for a short period (three years) and, therefore, further research is recommended to substantiate these findings.

## Conclusions

The main factors that facilitate saving with saving groups include awareness about the importance of saving, benefits of saving with saving groups such as easy acess to loans for meeting personal needs and health-related emergencies such as transportation for emergency obstetric care and safe custody of money. The factors that constrain saving include poverty and poor management practises. Overall however this work suggests that ‘Saving groups are like a sprouting bud that needs to be nurtured rather than uprooted’, because they seem to have the potential to act as a safety net for poor communities that have no health insurance. Implementers of maternal and newborn programmes should therefore encourage households to save using saving groups in addition to other methods. This will require increased sensitization about the importance of saving as a means of preparing for birth, in addition to the benefits of saving through a saving group. Such sensitization efforts should specifically target men, who tended to prioritize other things over maternal health. Local governments should therefore strengthen the management capacity of saving groups so as to ensure their efficient running through partnerships with NGOs that can provide support to such groups.

## Paper context

There is a dearth of literature about the use of saving groups to support birth preparedness. Our work shows that saving through saving groups enhanced the ability of households to meet financial costs at the time of birth and provided a safety net during emergencies. More effort should be directed towards increasing awareness about the benefits of saving for maternal health through saving groups and strengthening the management of these saving groups.
